# Controllability of Deterministic Networks with the Identical Degree Sequence

**DOI:** 10.1371/journal.pone.0127545

**Published:** 2015-05-28

**Authors:** Xiujuan Ma, Haixing Zhao, Binghong Wang

**Affiliations:** 1 School of Computer Science, Shaanxi Normal University, Xi’an, Shaanxi, 710062, P. R. China; 2 School of Computer Science, Qinghai Normal University, Xining, Qinghai, 810008, P. R. China; 3 Department of Modern Physics, University of Science and Technology of China, Hefei, Anhui, 230026, P. R. China; Nankai University, CHINA

## Abstract

Controlling complex network is an essential problem in network science and engineering. Recent advances indicate that the controllability of complex network is dependent on the network's topology. Liu and Barabási, et.al speculated that the degree distribution was one of the most important factors affecting controllability for arbitrary complex directed network with random link weights. In this paper, we analysed the effect of degree distribution to the controllability for the deterministic networks with unweighted and undirected. We introduce a class of deterministic networks with identical degree sequence, called (*x*,*y*)-flower. We analysed controllability of the two deterministic networks ((1, 3)-flower and (2, 2)-flower) by exact controllability theory in detail and give accurate results of the minimum number of driver nodes for the two networks. In simulation, we compare the controllability of (*x*,*y*)-flower networks. Our results show that the family of (*x*,*y*)-flower networks have the same degree sequence, but their controllability is totally different. So the degree distribution itself is not sufficient to characterize the controllability of deterministic networks with unweighted and undirected.

## Introduction

Controlling is one of the most challenging problems in modern network science and engineering of complex network. In the past decades significant efforts have been devoted to understanding the controllability of the complex network [1–21]. A networked system is controllable if imposing appropriate external signals on a subset of its nodes, the system can be driven from any initial state to any final state in finite time [22–30]. The minimal set of driver nodes required to control a network is called the minimum driver node set (MDS). The minimum number of the driver nodes denoted by *N*_*D*_. Moreover, we can measure the controllability of a network by the *n*_*D*_, $n_{D}=\frac{N_{D}}{N}$. Lin proposed the structural controllability theory in 1974 [24] and gave two factors influencing the structural controllability. He proposed minimal structure that ensured the structural controllability. However, the structural controllability theory didn’t solve the problem of minimal set of driver nodes. Liu, Slotine and Barabási [7] studied the controllability of various random directed networks and proposed a method to get *N*_*D*_. Recently Yuan and Zhao et.al [11] introduced the exact-controllability framework based on the maximum multiplicity to identify the *N*_*D*_ of arbitrary network topology. For more complicated model networks and many real-word weighted networks with distinct node-degree distributions, the exact controllability can be efficiently assessed by numerical computations. In spite of their results offered a general tool to solve the *N*_*D*_, we are more interested in the influence of network topologies to controllability. To find the factors which affect the controllability of the complex network, researchers from different perspectives were in the study of controllability [1–18]. In previous research, one of the most significant conclusions was given by Liu, Slotine and Barabási [7]. Their main conclusion is that the controllability of random directed network is determined by the degree distributions of network. That means, for arbitrary random directed networks, if they had different degree distribution then had different controllability. Meanwhile, the results be obtained were base on the random directed networks. Random network has most of the characteristics of the real network, however as Barabási said [31], it is hard to gain a visual understanding for many properties, and how do different nodes relate to each other. Deterministic networks are abundant in many real systems, such as power networks, computer networks, neural network and chemical network. Therefore, it is significant to study the controllability of deterministic networks. Li and Yuan et.al [17] analysed the controllability of several deterministic networks and gained the exactly expression of *N*_*D*_.

So far, we did not see relevant results in the controllability of deterministic complex networks with identical degree distributions. If two networks with same degree distribution, it is worthy of studying whether they have same controllability. On the other hand, there is an interesting problem as to whether the degree distribution is the only ingredient responsible for the controllability in the deterministic networks with unweighted and undirected.

To analyse the controllability of deterministic networks with identical degree distribution, we introduce the family of deterministic networks with fractal feature, which was called (*x*, *y*)-flower [32, 33] and has identical degree sequence. In the field of complex networks, fractal properties and self-similarities are shared by many real network systems [34–37] include World-Wide-Web, biological and social networks. In a lots of fractal networks, the (*x*, *y*)-flower networks displayed some remarkable properties such as scale-free, non-clustered and their degree distribution follows the power-law distribution. On the other hand, the (*x*, *y*)-flower has small world property for *x* = 1 and has large world property for *x* = *y*. Hence, it is worthwhile to investigate the dynamic processes of the (*x*, *y*)-flower networks. Zhang et.al [38] studied the percolation of two networks in (*x*, *y*)-flower networks, which were (1, 3)-flower network and (2, 2)-flower network respectively. Their results indicated that power-law degree distribution alone didn’t suffice to characterize the percolation threshold on (1, 3)-flower and (2, 2)-flower under bond percolation. Lin et.al [33] studied the spanning trees of (*x*, *y*)-flower networks, the authors found that the entropy of spanning trees on (*x*, *y*)-flower networks were different although the networks had the identical degree distribution. Thus, the degree distribution alone was not sufficient enough to describe the spanning trees of the network.

In this paper, we analysed the controllability and give accurate results of the minimum number of driver nodes for (1, 3)-flower networks and (2, 2)-flower network by exact controllability theory. Our results show that although (1, 3)-flower and (2, 2)-flower have same degree sequence, their controllability are totally different; that is, the *N*_*D*_ of the (1, 3)-flower is about half of the (2, 2)-flower. Furthermore, we verify the results in (*x*, *y*)-flower networks by computer simulation. Simulation results show that the (*x*, *y*)-flower networks of *x* = 1 have the identical degree sequence with *x* = *y*, but their controllability are entirely different. So the degree distribution itself is not sufficient to characterize the controllability of deterministic networks with unweighted and undirected.

## Model

### Model and properties of (*x*, *y*)-flower

In order to analyse the controllability of the (*x*, *y*)-flower networks, we first introduce the construction and structural properties of the (*x*, *y*)-flower. The (*x*, *y*)-flower are built in an iterative way. We denote the (*x*, *y*)-flower after *t* generations of evolution by *F*_*t*_(*x*, *y*)(*n* ≥ 0), without loss of generality we assumed that *x* ≤ *y* and *y* > 1. The (*x*, *y*)-flower are constructed by the algorithm as follows [32, 33]:

1. At the initial time *t* = 0, *F*_0_(*x*, *y*) is an edge connecting two node, called the initial nodes.
2. After, for *t* ≥ 1, *F*_*t*_(*x*, *y*) is derived from the *F*_*t*−1_(*x*, *y*). For *x* = 1, every old edge generates *y* − 1 new nodes, and all of these *y* − 1 new nodes and the old edge form a circle of length *y*+1. For *x* > 1, every old edge connecting two old nodes is removed and replaced by two parallel paths with the two old nodes as the ends of the two parallel paths: the above *y* − 1 nodes and the two old nodes form a path of length *y*, while the below *x* − 1 nodes and the two old nodes constitute another path of length *x*.

**Fig 1:**
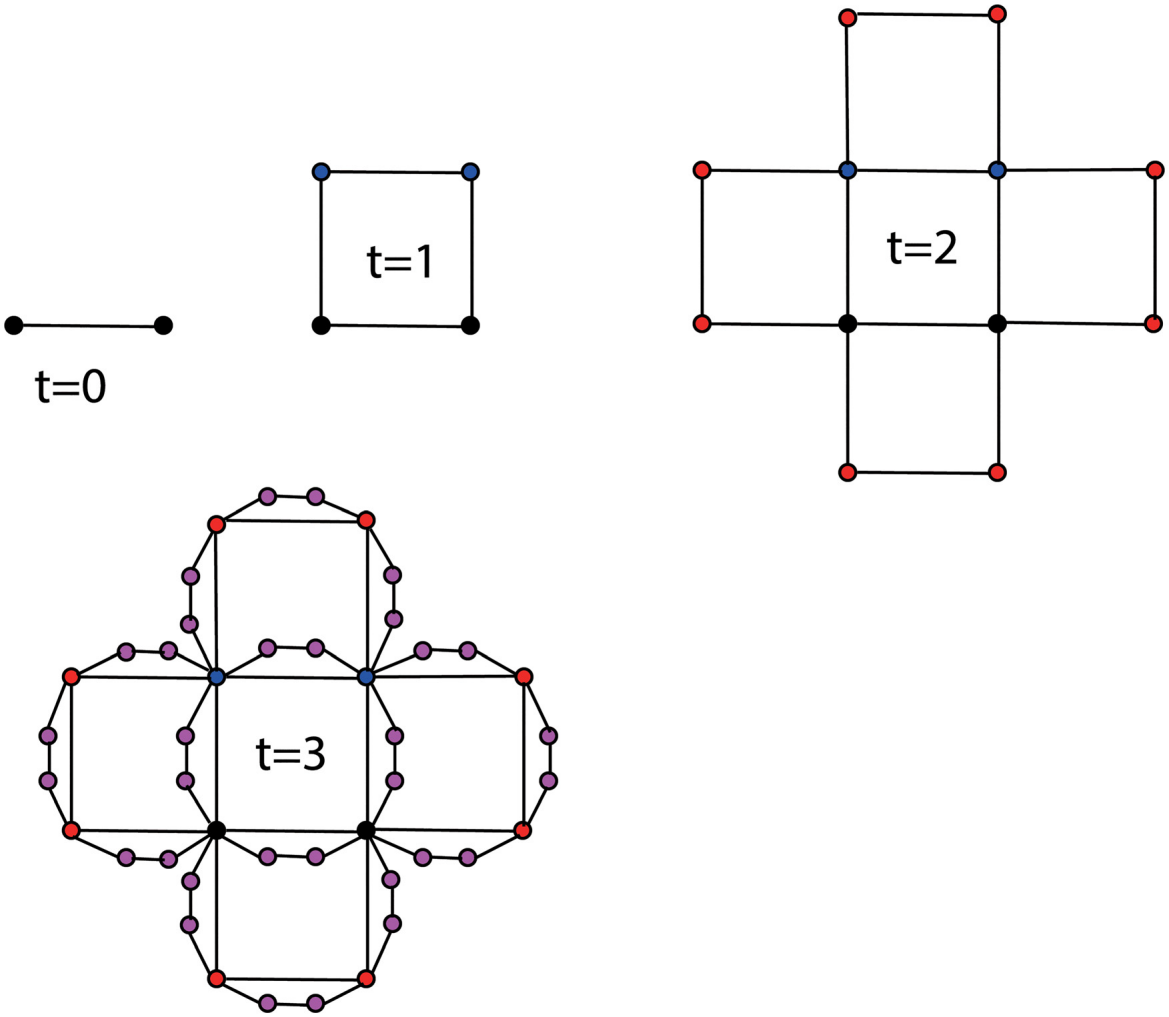
Illustration of the growing process for the (1, 3)-flower. Where, every old edge generates 2 new nodes. All of these 2 new nodes and the old edge form a circle of length 4.

**Fig 2:**
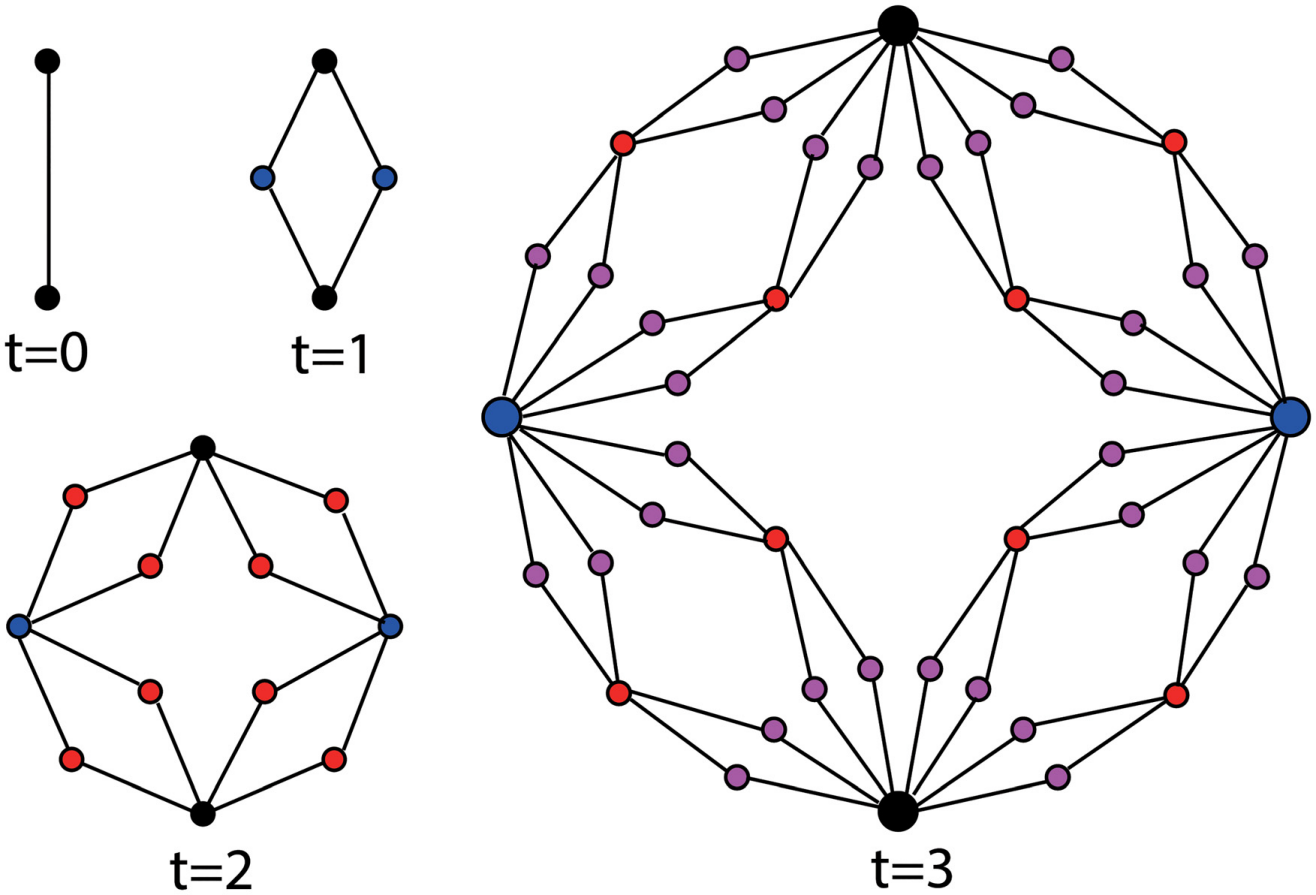
Illustration of the growing process for the (2, 2)-flower. Where, each old edge connecting two old nodes is removed and replaced by two pathes with the two old nodes as the ends; one new node and the two old nodes form one path of length 2, while another new node and the two old nodes constitute the other path of length 2.

According the constructed process of (*x*, *y*)-flower, we can obtain some topological properties [32, 33] as follows:

1. Let *E*_*t*_ is the number of edges and *N*_*v*_(*t*) is the number of total nodes in *F*_*t*_(*x*, *y*), then it is easy to calculate *E*_*t*_ = (*x* + *y*)^*t*^ and $N_{v}(t)=\frac{x+y-2}{x+y-1}{\left(x+y\right)}^{t}+\frac{x+y}{x+y-1}$.
2. Since the *F*_*t*_(*x*, *y*) is deterministic construction, we obtain the degree sequence of *F*_*t*_(*x*, *y*). In *F*_*t*_(*x*, *y*), the degree of node *i* at time *t* denoted by *k*_*i*_(*t*). By construction, the degree *k*_*i*_(*t*) evolves with time as *k*_*i*_(*t*) = 2*k*_*i*_(*t*−1) = 2^*s*^(*s* = 1, 2, …, *t*), that is, the degree of node *i* increases by a factor 2 at each time step. Let *N*_*t*_(*s*)(*s* = 1, 2, …, *t*) be the number of nodes with degeree 2^*s*^(*s* = 1, 2, …, *t*) in *F*_*t*_(*x*, *y*), we have:
  $$\begin{array}{c} N_{t}\left(s\right)=\{\begin{array}{cc} \left(x+y-2\right){\left(x+y\right)}^{t-s}, & s<t;\\ x+y, & s=t. \end{array} \end{array}$$ (1)
  Thus, all the class of *F*_*t*_(*x*, *y*) with the same *x* + *y* have the identical degree sequence.
3. The degree distributions of (*x*, *y*)-flower obey a power-law distribution *P*(*k*) ∼ *k* ^−*γ*^, with the exponent $\gamma =1+\frac{\text{ln}(x+y)}{\text{ln}2}$. According the algorithm of constructing the (*x*, *y*)-flower, we obtain the (1, 3)-flower and the (2, 2)-flower after *t* iterations, and denoted by *F*_*t*_(1, 3) and *F*_*t*_(2, 2) respectively. Figs 1 and 2 respectively show the growth process of the (1, 3)-flower and the (2, 2)-flower.

For these two networks, after *t* iterations, we have *E*_*t*_ = 4^*t*^, $N_{v}(t)=\frac{2}{3}(4^{t}+2)$. Let *L*_*v*_(*t*) be the number of nodes generated at step *t*. Each existing edge at a given step will yield two new nodes at the next step, this leads to *L*_*v*_(*t*) = 2*E*_*t*−1_ = 2×4^*t*−1^(*t* ≥ 1).

**Fig 3:**
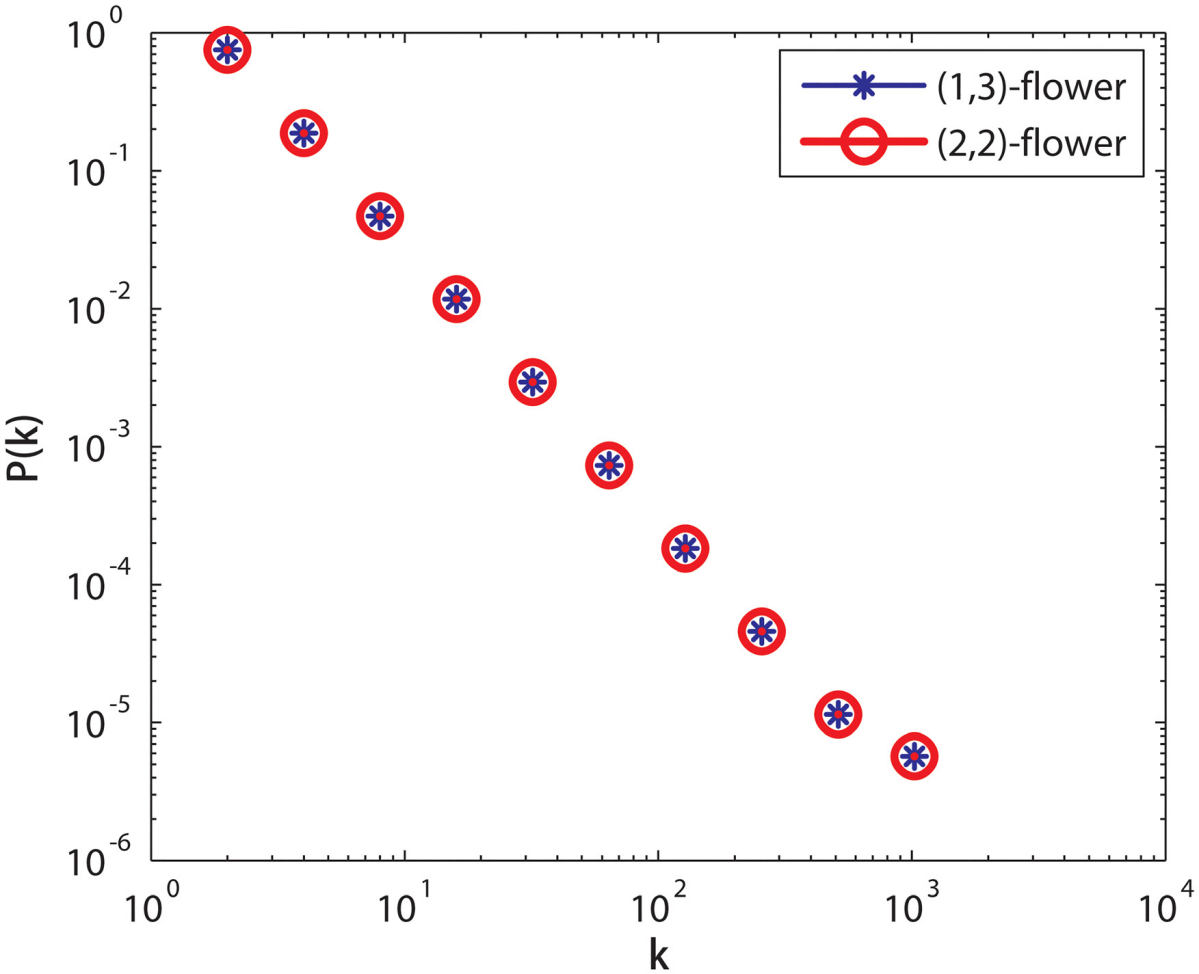
The degree distribution of *F*_*t*_(1, 3) and *F*_*t*_(2, 2). Two networks have identical degree distribution and their degree distribution obey a power-law degree distribution *P*(*k*) ∼ *k* ^−3^.

The average node degree after *t* iterations is $\langle k_{t}\rangle =\frac{2E_{t}}{N_{v}(t)}=\frac{3\times 4^{t}}{4^{t}+2}$, which approaches 3 for large *t*. Meanwhile, the two networks have an identical degree sequence, and they obey a power-law degree distribution *P*(*k*) ∼ *k* ^−3^. Fig 3 show that the degree distributions of *F*_*t*_(1, 3) network and *F*_*t*_(2, 2) network. From this figure, we can see that the degree distributions of the *F*_*t*_(1, 3) and *F*_*t*_(2, 2) are identical.

In this paper, we analysed the controllability of (1, 3)-flower and (2, 2)-flower by the exact controllability theory. Next, let’s start by reviewing some basic concepts of the exact controllability theory.

### Exact controllability theory

One controlled complex network with *N* nodes, described by the following linear ordinary differential equations [7, 11]

$$\begin{array}{c} \dot{X}=Ax+Bu, \end{array}$$ (2)

where the vector **x** = (*x*_1_, *x*_2_, ⋯, *x*_*N*_)^*T*^ stands for the states of *N* nodes, *A* ∈ *R* ^*N* × *N*^ denotes the coupling matrix of a complex network, **u** is the vector of *m* controllers: **u** = (*u*_1_, *u*_2_, ⋯, *u*_*m*_)^*T*^ and *B* is the *N* × *m* input matrix. Note that each node is captured by single state, saying one-dimensional nodal dynamics. In order to fully control the complex network, we should choose right *B* and **u**. Our purpose is to devise a matrix *B*, corresponding to the minimum number of input signals imposing on minimum set of driver nodes. According to the results of Liu [7] and Yuan [11],

$$\begin{array}{c} N_{D}\equiv min\left\{rank\left(B\right)\right\}. \end{array}$$ (3)

According to the PBH rank condition [23, 28–30] Yuan et.al [11] proved that for arbitrary network, the minimum number *N*_*D*_ of controllers or drivers are determined by the maximum geometric multiplicity *μ*(*λ*_*i*_) of the eigenvalue *λ*_*i*_ of *A*:

$$\begin{array}{c} N_{D}=\max\left\{\mu \left(\lambda_{i}\right)\right\}, \end{array}$$ (4)

where *μ*(*λ*_*i*_) = *dimV*_*λ*_*i*__ = *N*−*rank*(*λ*_*i*_ *I*_*N*_−*A*) and *λ*_*i*_(*i* = 1, ⋯, *l*) are the distinct eigenvalues of *A*. For a symmetric coupling matrix, its geometric multiplicity equals to algebraic multiplicity. For an undirected networks, *N*_*D*_ is determined by the maximum algebraic multiplicity *δ*(*λ*_*i*_) of *λ*_*i*_ [11]:

$$\begin{array}{c} N_{D}=max\left\{\delta \left(\lambda_{i}\right)\right\}. \end{array}$$ (5)

For a large sparse network with a small fraction of self-loops, in which the number of links scales with *N* in the limit of large *N* [39], *N*_*D*_ is simply determined by the rank of the coupling matrix *A* [11]

$$\begin{array}{c} N_{D}\equiv max\left\{1,N-rank\left(A\right)\right\}, \end{array}$$ (6)

which means the eigenvalue 0 has a maximum multiplicity.

For a densely connected network with a small fraction of self-loop in which the zeros in *A* same scale with *N* in the limit of large *N*, we have [11]

$$\begin{array}{c} N_{D}\equiv max\left\{1,N-rank\left(wI_{N}+A\right)\right\}. \end{array}$$ (7)

The measure of controllability denoted by *n*_*D*_, it is defined as the ratio of *N*_*D*_ to the network size *N* [7]:

$$\begin{array}{c} n_{D}=\frac{N_{D}}{N}. \end{array}$$ (8)

Using the Eqs (3)–(7) we can calculate *N*_*D*_ of an arbitrary network topology. For the deterministic (1, 3)-flower network and (2, 2)-flower network, we obtain the exact solutions of *N*_*D*_ in (1, 3)-flower network and (2, 2)-flower network by equation Eq (6). Moreover, we get the measure of controllability *n*_*D*_ in (*x*, *y*)-flower by equation Eq (8).

## Analysis

We derive the formula of *N*_*D*_ and *n*_*D*_ of *F*_*t*_(1, 3) and *F*_*t*_(2, 2) from network topology by exact controllability theory. To calculate the eigenvalues of fractal scale-free networks, Zhang and Hu et.al introduced a method [36] Inspired by this method, we calculate the rank of the adjacency matrix in *F*_*t*_(1, 3) and *F*_*t*_(2, 2).

### The controllability of (1, 3)-flower network

Let *α* be the set of nodes of the network at the *s*-step, *α* = *N*_*s*_; *β* be the set of nodes that are generated at the (*s* + 1)-th iteration, *β* = *N*_*s*+1_−*N*_*s*_. The matrix *A*_*s*+1_(1, 3) is the adjacency matrix of *F*_*s*+1_(1, 3), which has the following block form:

$$\begin{array}{c} A_{s+1}\left(1,3\right)=(\begin{array}{cc} A_{\alpha ,\alpha } & A_{\alpha ,\beta }\\ A_{\beta ,\alpha } & A_{\beta ,\beta } \end{array}). \end{array}$$ (9)

The matrix *A*_*α*,*α*_ is the adjacency matrix of (1, 3)-flower at *t* = *s*, which represents the adjacency relations of old nodes. The matrix *A*_*β*,*β*_ represents the adjacency relationships of new nodes, and the matrix $A_{\beta ,\alpha }=A_{\alpha ,\beta }^{T}$ characterizes the relationships between new nodes and old nodes. By the construction of the *F*_*s*+1_(1, 3) and appropriately implementing elementary transformations for the matrix *A*_*s*+1_(1, 3), we get

$$\begin{array}{c} A_{s+1}\left(1,3\right)∼(\begin{array}{cc} 0 & 0\\ 0 & I_{\beta ,\beta } \end{array}), \end{array}$$ (10)

where the *I*_*β*,*β*_ is an identity matrix with *β* rows and *β* columns. For details on elementary transformations, see Supporting Information(S1 File). Obviously, *rank*(*A*_*s*+1_) = *rank*(*I*_*β*,*β*_) = *β* = *N*_*s*+1_−*N*_*s*_. Thus by Eq (6),

$$\begin{array}{ccc} N_{D}\left(F_{s+1}\left(1,3\right)\right) & = & \max\{1,N_{s+1}-rank\left(A_{s+1}\right)\}\\ & = & \max\{1,N_{s}\}. \end{array}$$

So,

$$\begin{array}{c} N_{D}\left(F_{t}\left(1,3\right)\right)=\{\begin{array}{cc} 1, & t=0;\\ \frac{2}{3}\left(4^{t-1}+2\right), & t\geq 1. \end{array} \end{array}$$ (11)

The measure of controllability *n*_*D*_ of *F*_*t*_(1, 3) network is obtained by Eq (8):

$$\begin{array}{c} n_{D}=\frac{N_{D}\left(F_{t}\left(1,3\right)\right)}{N_{t}}=\frac{\frac{2}{3}(4^{t-1}+2)}{\frac{2}{3}(4^{t}+2)}=\frac{4^{t-1}+2}{4^{t}+2} \end{array}$$ (12)

with its thermodynamic limit:

$$\begin{array}{c} \lim_{t\to \infty }n_{D}=\lim_{s\to \infty }\frac{4^{t-1}+2}{4^{t}+2}=\frac{1}{4}. \end{array}$$ (13)

### The controllability of (2, 2)-flower network

The controllability of the (2, 2)−*flower* network can be analysed in the same way as the (1, 3)−*flower* network. Similarly, let *α* be the set of nodes that are belong to the s-step network *F*_*t*_(2, 2); and *β* be the set of nodes that are generated at the (*s* + 1)-th iteration, where the value of *α* and *β* are same with *F*_*t*_(1, 3). From the construction, at (*s* + 1)-th step, the adjacency matrix *B*_*s*+1_(2, 2) of the network *F*_*s*+1_(2, 2) has the following form:

$$\begin{array}{ccc} B_{s+1} & = & \left(\begin{array}{cc} B_{\alpha ,\alpha } & B_{\alpha ,\beta }\\ B_{\beta ,\alpha } & B_{\beta ,\beta } \end{array}\right)\\ & = & \left(\begin{array}{cc} 0 & B_{\alpha ,\beta }\\ B_{\beta ,\alpha } & 0 \end{array}\right)\\ & = & (\begin{array}{cc} 0 & B_{\alpha ,\beta }\\ B_{\alpha ,\beta }^{T} & 0 \end{array}). \end{array}$$ (14)

Then, we can obtain the rank of the adjacency matrix *B*_*s*+1_ as:

$$\begin{array}{ccc} rank(B_{s+1}) & = & rank(B_{\alpha ,\beta })+rank(B_{\alpha ,\beta }^{T})\\ & = & 2rank(B_{\alpha ,\beta }). \end{array}$$ (15)

By calculating(S1 File) we obtain *rank*(*B*_*α*,*β*_) = *N*_*s*_ − 1. Then, by Eq (15), *rank*(*B*_*s*+1_) = 2(*N*_*s*_−1). According the Eq (6), we have:

$$\begin{array}{c} N_{D}\left(F_{t}\left(2,2\right)\right)=\{\begin{array}{cc} 1, & t=0;\\ \frac{2}{3}\left(2\times 4^{t-1}+1\right), & t\geq 1. \end{array} \end{array}$$ (16)

Furthermore, the measure of controllability *n*_*D*_ of *F*_*t*_(2, 2) network is obtained:

$$\begin{array}{c} n_{D}=\frac{N_{D}\left(F_{t}\left(2,2\right)\right)}{N_{t}}=\frac{\frac{2}{3}(2\times 4^{t-1}+1)}{\frac{2}{3}(4^{t}+2)}=\frac{2\times 4^{t-1}+1}{4^{t}+2} \end{array}$$ (17)

with its thermodynamic limit:

$$\begin{array}{c} \lim_{t\to \infty }n_{D}=\lim_{t\to \infty }\frac{2\times 4^{t-1}+1}{4^{t}+2}=\frac{1}{2}. \end{array}$$ (18)

### Numerical analysis

Lots of results about controllability of the complex network show that the controllability is mainly determined by the degree distribution of the network. We numerically analysed the controllability of a class of deterministic networks with the identical degree sequence. However from our results, the family of deterministic networks with identical degree sequence have different controllability.

**Fig 4:**
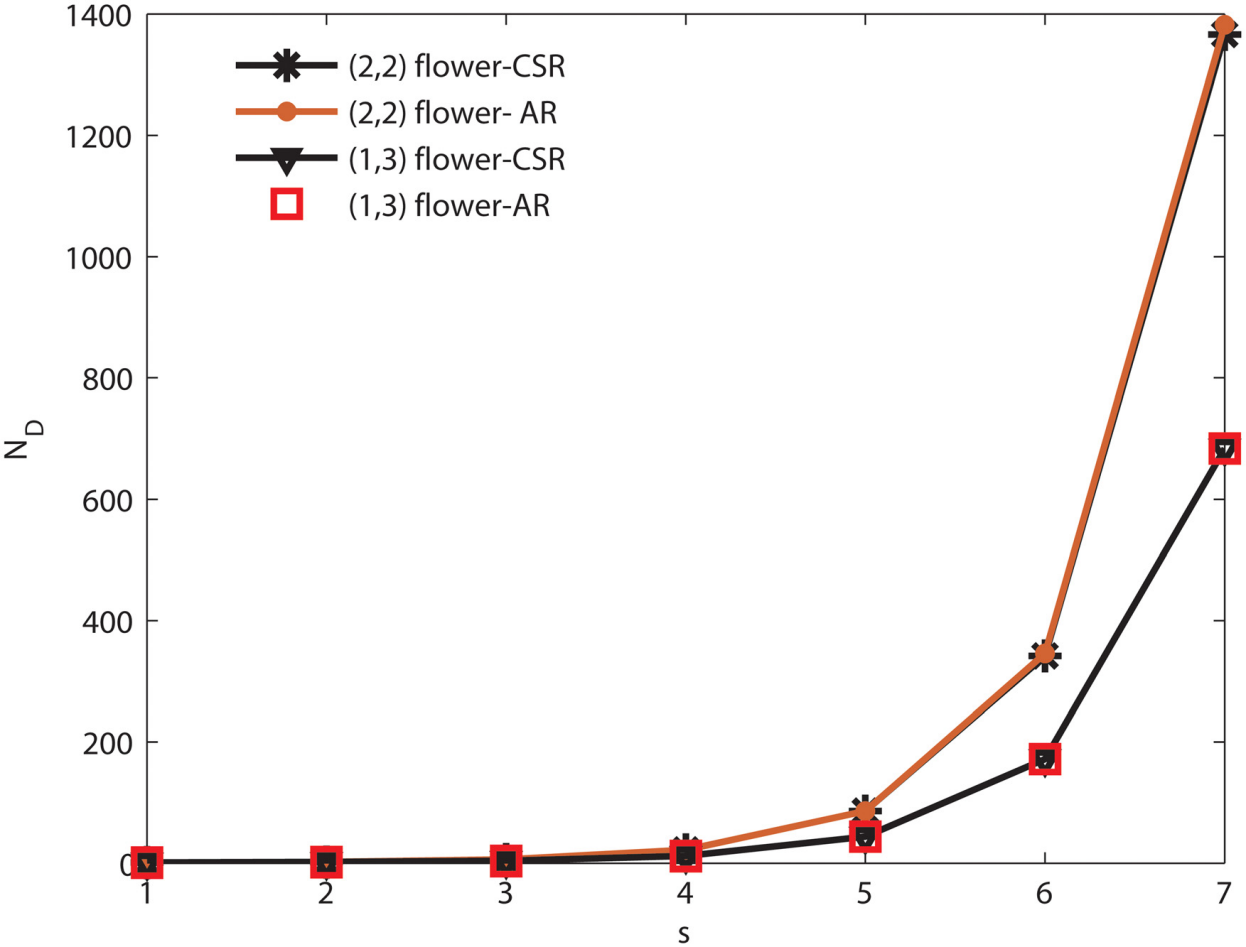
The minimum number of driver nodes *N*_*D*_ of the two networks increased with *s*. CSR denotes the results by computer simulation. AR denotes the results predicted by Eqs (11) and (16) in two networks. Two networks are iterated to step 7. For the two networks, the CSR and AR are exactly same, respectively. But the *N*_*D*_ of the (2, 2)-flower network is more than the (1, 3)-flower network.

**Fig 5:**
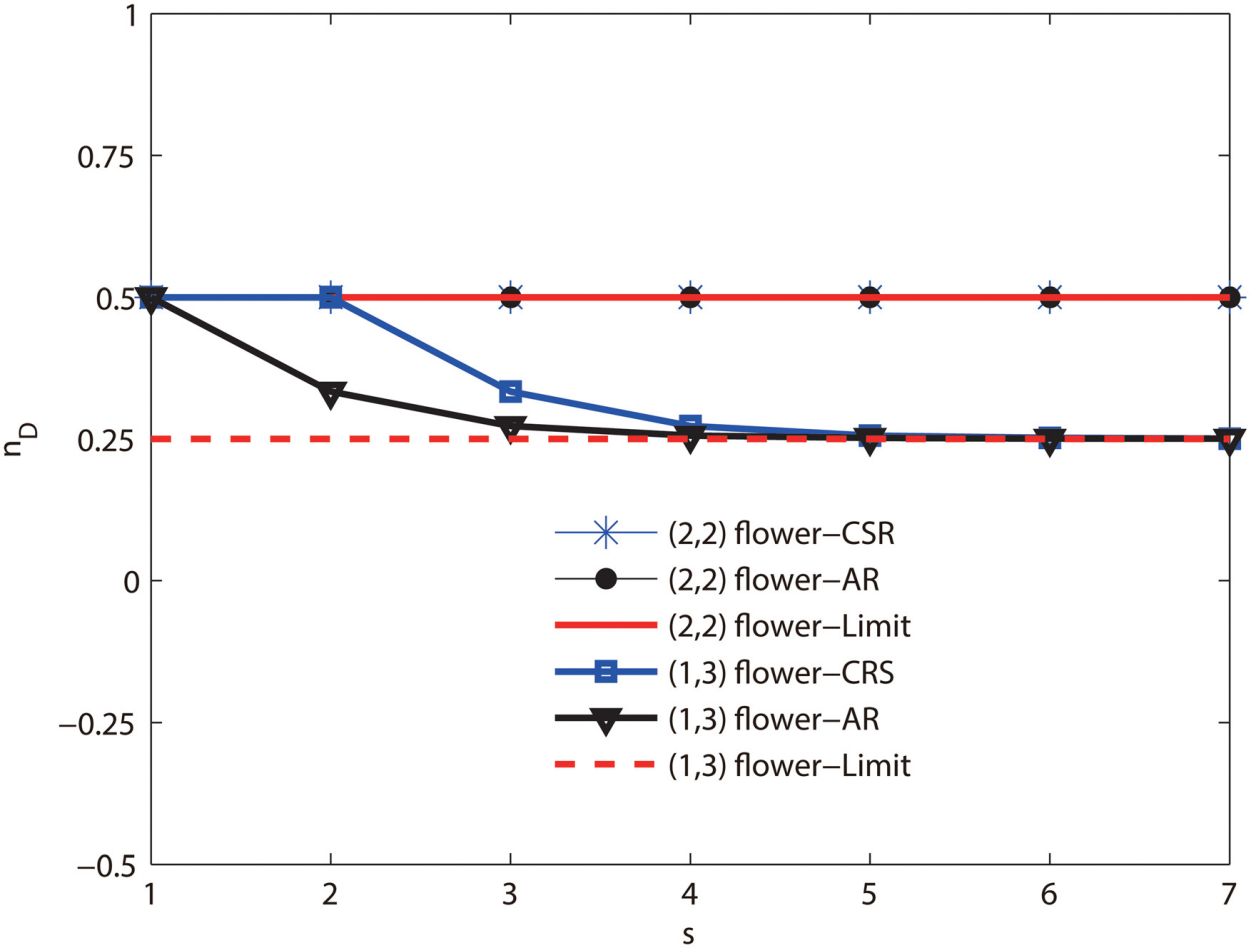
Controllability measure *n*_*D*_ is a function of iteration step *s* for the (2, 2) flower network and the (1, 3)-flower network, respectively. CSR denotes the results by computer simulation. AR denotes the results predicted by Eqs (12) and (17) for the two networks. Limit denotes the thermodynamic limit predicted by Eqs (13) and (18). The two networks are iterated to step 7. For the (2, 2)-flower network, three curves of CRS, AR and Limit are coincide. For the (1, 3) flower network, three curves of CRS, AR and Limit are almost entirely the same at *s* ≥ 4.

**Numerical analysis of (1, 3)-flower network and (2, 2)-flower network** As shown in Fig 4, we calculate *N*_*D*_ of (1, 3)-flower network and (2, 2)-flower network by computer numerical simulation, and compare with the theory results of Eqs (11) and (16). We see that the computer numerical simulations are in accordance with theory results obtained from equation. On the other hand, as discussed above, the minimum number *N*_*D*_ of driver nodes of the two networks increases exponentially as the iteration step *t* increase, as reflected in Eqs (11) and (16). Moreover, Fig 4 shows that the *N*_*D*_ of the two networks are totally different. The size of *N*_*D*_ in the (2, 2)-flower network is more than the (1, 3)-flower network. This indicates that the (1, 3)-flower network is easier to control. The Fig 5 shows both limits of the two networks’ controllability measure *n*_*D*_ converge to the constant lower than 1, as predicted by Eqs (12), (13), (17) and (18). The controllability measure *n*_*D*_ of the (1, 3)-flower network falls faster and tends to a constant $\frac{1}{4}$, and the *n*_*D*_ of the (2, 2)-flower network tends to $\frac{1}{2}$. It shows that the (1, 3)-flower network is much more easily controlled.

**Fig 6:**
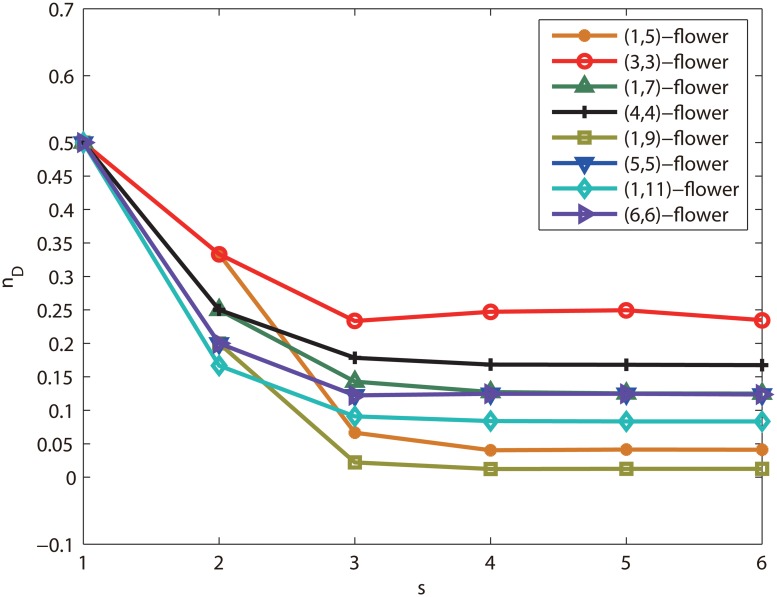
Controllability measure *n*_*D*_ of the (*x*, *y*) flower networks at different iteration *s*. Controllability measure *n*_*D*_ is a function of iteration step *s* for the (*x*, *y*) flower networks, where we take *x*+*y* = 6, 8, 10 and 12 respectively. These networks are iterated to step 5. The controllability measure are equal of all networks at *s* = 0, after start to fall at *s* > 0, and converges a constant lower than 1 at *s* > 4.

**Fig 7:**
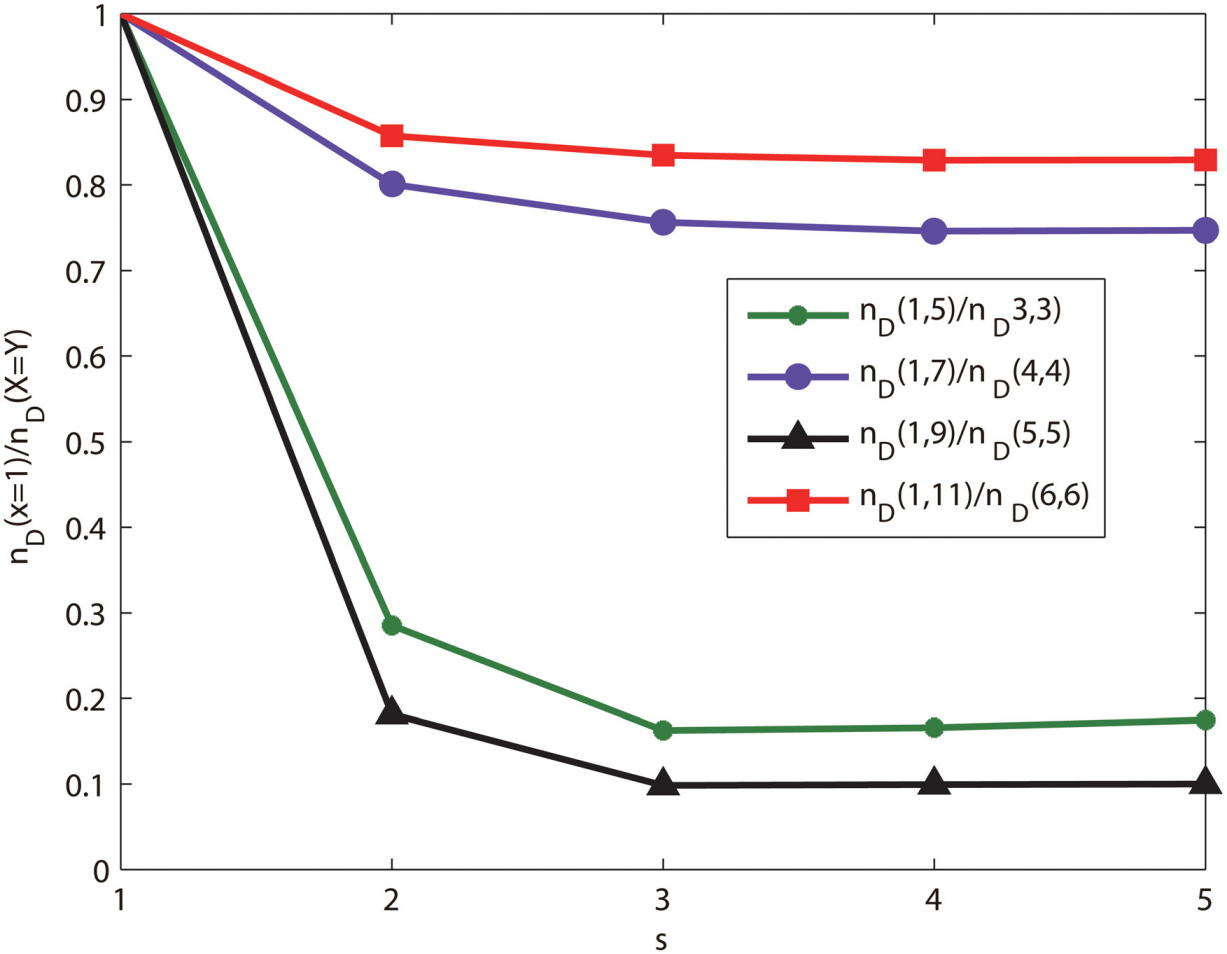
The ratio of the *n*_*D*_ for *x* = 1 and *x* = *y*. All ratios both are lower than 1.

**Numerical analysis of (*x*, *y*)-flower networks** In order to verify that a class of network with the identical degree sequence have different controllability, we give the simulation results of (*x*, *y*)-flower networks. We compare the controllability of (*x*, *y*)-flower networks, and obtain the same conclusions with (1, 3)-flower networks and (2, 2)-flower network. That is, for (*x*, *y*)-flower networks, the networks of *x* = 1 have the identical degree distribution with the networks at *x* = *y*, but their controllability are different. Fig 5 shows that the controllability measure of networks for *x* = 1 and *x* = *y*. In the simulation, we give the controllability measure *n*_*D*_ with fixed *x*+*y* = 6, 8, 10 and 12, respectively. Fig 6 shows that the *n*_*D*_ of each network tends a constant lower than 1, and the controllability of each other is different. Furthermore, Fig 6 shows that the ratios of *n*_*D*_ for the network with *x* = 1 and *x* = *y*, all ratios are lower than 1. According to the results of Fig 7, the networks of *x* = 1 is controlled easy than the networks of *x* = *y*.

## Conclusions and discussion

In this paper, we investigate the controllability of a class of deterministic networks with the identical degree sequence. The family of (*x*, *y*)-flower networks are deterministic networks and have the same degree sequence. They have many good properties such as scale-free, non-clustered; the networks of *x* = 1 are small-world, but the networks of *x* = *y* are large world. Hence, it is worthwhile to investigate the processes taking place upon the model to find the different impact on dynamic processes. The family of (*x*, *y*)-flower networks are sparse deterministic networks with fractal characteristics, according the exact controllability theory, their controllability are completely determined by the rank of their adjacency matrix of the network. Due to the self-similarity of networks, we derived an exact expression of the minimum driver nodes for the (1, 3)-flower network and the (2, 2)-flower network by exactly controllability theory. Theoretical analysis and simulation results show that though the (1, 3)-flower network and (2, 2)-flower network have the identical degree sequence, their controllability are entirely different. Meanwhile, by comparing the controllability measure of (*x*, *y*)-flower networks, we find that the degree distributions of the networks *x* = 1 are identical with *x* = *y*, but they have different controllability. Moreover, the networks of *x* = 1 are display better controllability than the networks of *x* = *y*. The results indicate that the degree distribution alone is not sufficient to characterize the controllability of deterministic scale-free networks with unweight and undirect. Then, an important question arises: For the unweighted and undirected networks, which structural property has the decisive effect on the controllability in network with identical degree sequence? In the Ref [33] the authors studied the thresholds of bond percolation in the (*x*, *y*)-flower networks, which indicated that the thresholds of bond percolation of the networks *x* = 1 are smaller than the *x* = *y*. In recent years, some new results about the entropy of graphs are obtained [29, 32, 38, 40–42]. In the Ref [32, 38] the authors studied the entropy of the spanning trees of two networks, and they got similar results that the entropy of the spanning trees in the networks *x* = 1 are smaller than *x* = *y*. Thus, it is a worthwhile to studying the relation between the controllability, the thresholds and the spanning trees.

## Supporting Information

S1 File This file includes the process how to calculate the rank of the adjacency matrix in *F*_*t*_(1, 3) network and *F*_*t*_(2, 2) network. (PDF)
