# Supplementary material for: Controllability of Deterministic Networks with the Identical Degree Sequence
Source: PLoS One. 2015 May 28;10(5):e0127545. doi: 10.1371/journal.pone.0127545 (PMC4447402; doi:10.1371/journal.pone.0127545)
Supplement: S1 File — (PDF) [file pone.0127545.s001.pdf]

Controllability of deterministic networks with the identical degree sequence

Supporting Information

Computation the matrix rank of $F_t(1,3)$ network

Let α be the set of nodes of the network at the s-step, and β be the set of nodes that are generated at the $(s+1)$-th iteration. The matrix $A_{s+1}(1,3)$ is the adjacency matrix of $F_{s+1}(1,3)$, which has the following block form:

$$A_{s+1}(1,3) = \begin{pmatrix} A_{\alpha,\alpha} & A_{\alpha,\beta} \\ A_{\beta,\alpha} & A_{\beta,\beta} \end{pmatrix}. \quad (1)$$

By the construction of the $F_{s+1}(1,3)$, for each edge at s-th, there will be two new nodes attached to ends of each edge at $(s+1)$-th, and one edge between two new nodes. The matrix $A_{\alpha,\alpha}$ is the adjacency matrix of $(1,3)$-flower at $t=s$, which represents the adjacency relations of old nodes. When a node is added to the network at s, then its degree is 2. With the evolution of network, at $t=s$, the degree of nodes added at first step is 2^s, the degree of nodes added at second step is 2^{s-1}, and the degree of nodes added at ith step is $2^{(s-i+1)}$. Thus, in the matrix $A_{\alpha,\alpha}$, the number of 1 in each column (form 1-th column to N_1-th column) is 2^s; the number of 1 in each column(from column N_i+1 to column N_{i+1}), $(i=1,2,\cdots,s-1)$ is 2^{s-i}. The matrix $A_{\beta,\beta}$ represents the adjacency relationships of new nodes, and the matrix $A_{\beta,\alpha}=A^T_{\alpha,\beta}$ characterizes the relationships between new nodes and old nodes. At $s+1$ step, the matrix $A_{\alpha,\beta}$ have some structural features as following:
1. The old nodes with label as 1 to N_1 generated 2^s new nodes, that means, each row from 1-th row to N_1-th row of the matrix $A_{\alpha,\beta}$ has 2^s 1, respectively.
2. The old nodes with label as N_i+1 to N_{i+1} genetated 2^{s-i} new nodes, that means, there are 2^{s-i} 1 in N_i+1-th row to N_{i+1}-th row, where, $i=1,2,\cdots,s-1$. Now, we appropriately exchange some columns in the matrix $A_{\alpha,\beta}$, in order to put these elements with value 1 together of each row, then the matrix $A_{\alpha,\beta}$ transformed into the matrix $M_{\alpha,\beta}$ and its form as follow:

$$M_{\alpha,\beta} = \begin{pmatrix}
1\cdots1 & 0\cdots0 & \cdots & 0\cdots0 & 00\cdots & 00 \\
0\cdots0 & 1\cdots1 & \cdots & 0\cdots0 & 00\cdots & 00 \\
\cdots & \cdots & \cdots & \cdots & \cdots & \cdots \\
0\cdots0 & 0\cdots0 & \cdots & 1\cdots1 & 00\cdots & 00 \\
\cdots & \cdots & \cdots & \cdots & \cdots & \cdots \\
0\cdots0 & 0\cdots0 & \cdots & \cdots & 11\cdots & \cdots \\
\cdots & \cdots & \cdots & \cdots & \cdots & \cdots \\
0\cdots0 & 0\cdots0 & \cdots & \cdots & \cdots11 & 00 \\
0\cdots0 & 0\cdots0 & \cdots & 0\cdots0 & \cdots00 & 11
\end{pmatrix}.$$

Then, we implement same exchange for rows of the matrix $A_{\beta,\alpha}$, which transformed into the matrix $M_{\beta,\alpha}$ as following:

$$M_{\beta,\alpha} = \begin{pmatrix} 1 & 0 & \cdots & 0 & 0 & \cdots & 0 & 0 \\ \vdots & \vdots & \vdots & \vdots & \vdots & \vdots & \vdots & \vdots \\ 1 & 0 & \cdots & 0 & 0 & \cdots & 0 & 0 \\ 0 & 1 & \cdots & 0 & 0 & \cdots & 0 & 0 \\ \vdots & \vdots & \vdots & \vdots & \vdots & \vdots & \vdots & \vdots \\ 0 & 1 & \cdots & 0 & 0 & \cdots & 0 & 0 \\ \cdots & \cdots & \cdots & \cdots & \cdots & \cdots & \cdots & \cdots \\ 0 & 0 & \cdots & 1 & 0 & \cdots & 0 & 0 \\ \vdots & \vdots & \vdots & \vdots & \vdots & \vdots & \vdots & \vdots \\ 0 & 0 & \cdots & 1 & 0 & \cdots & 0 & 0 \\ \cdots & \cdots & \cdots & \cdots & \cdots & \cdots & \cdots & \cdots \\ 0 & 0 & \cdots & 0 & 1 & \cdots & 0 & 0 \\ 0 & 0 & \cdots & 0 & 1 & \cdots & 0 & 0 \\ \cdots & \cdots & \cdots & \cdots & \cdots & \cdots & \cdots & \cdots \\ 0 & 0 & \cdots & 0 & 0 & \cdots & 1 & 0 \\ 0 & 0 & \cdots & 0 & 0 & \cdots & 1 & 0 \\ 0 & 0 & \cdots & 0 & 0 & \cdots & 0 & 1 \\ 0 & 0 & \cdots & 0 & 0 & \cdots & 0 & 1 \end{pmatrix}$$

The number of 1 of each column in the matrix $M_{\beta,\alpha}$ equal to the same column in the matrix $A_{\alpha,\alpha}$.

According the construction of the $F_{s+1}(1,3)$, at $t = s+1$, there is one edge between two adjacency new nodes, so each column of the matrix $A_{\beta,\beta}$ has one 1, its form as following:

$$A(\beta,\beta) = \begin{pmatrix} 0 & 1 & 0 & 0 & \cdots & 0 & 0 \\ 1 & 0 & 0 & 0 & \cdots & 0 & 0 \\ 0 & 0 & 0 & 1 & \cdots & 0 & 0 \\ 0 & 0 & 1 & 0 & \cdots & 0 & 0 \\ \cdots & \cdots & \cdots & \cdots & \cdots & \cdots & \cdots \\ 0 & 0 & 0 & 0 & \cdots & 0 & 1 \\ 0 & 0 & 0 & 0 & \cdots & 1 & 0 \end{pmatrix}.$$

After exchanging some columns, the matrix $A_{\beta,\beta}$ convert into the matrix $M_{\beta,\beta}$, there is still one 1 in each column. Through above rows and columns exchange, the matrix $A_{s+1}(1,3)$ converts into the block form as following:

$$A_{s+1}(1,3) \sim \begin{pmatrix} A_{\alpha,\alpha} & M_{\alpha,\beta} \\ M_{\beta,\alpha} & M_{\beta,\beta} \end{pmatrix}. \quad (2)$$

For each row of the matrix $M_{\beta,\alpha}$ and $M_{\beta,\beta}$ multiply by (-1), then add each row of the matrix $-M_{\beta,\alpha}$ and $-M_{\beta,\beta}$ to the corresponding row of the matrix $A_{\alpha,\alpha}$ and $M_{\alpha,\beta}$, such that the adjacency matrix of $A_{s+1}(1,3)$ can be translated into the following form:

$$A_{s+1}(1,3) \sim \begin{pmatrix} 0 & 0 \\ -M_{\beta,\alpha} & -M_{\beta,\beta} \end{pmatrix}. \quad (3)$$

The transformation procedure is: let $A_{\alpha,\alpha} = (a_{p,q})_{\alpha\times\alpha}$, $-M_{\beta,\alpha} = (b_{p,q})_{\beta\times\alpha}$, if $a_{ij} = 1$, $b_{kj} = -1$, then add the k-th row of $-M_{\beta,\alpha})$ and $-M_{\beta,\beta}$ to the i-th row of $A_{\alpha,\alpha}$ and $M_{\alpha,\beta}$.

For the matrix $-M_{\beta,\alpha}$, we implement similarly elementary column transformation, then each element of the matrix $-M_{\beta,\alpha}$ is 0, so we have:

$$A_{s+1}(1,3) \sim \begin{pmatrix} 0 & 0 \\ 0 & M_{\beta,\beta} \end{pmatrix}. \tag{4}$$

Since each row of the matrix $M_{\beta,\beta}$ has only one element 1 and lie in different column, the matrix $M_{\beta,\beta}$ is through the rows exchange into an identity matrix $I_{\beta,\beta}$. Thus, we have

$$A_{s+1} \sim \begin{pmatrix} 0 & 0 \\ 0 & I_{\beta,\beta} \end{pmatrix}. \tag{5}$$

Obviously, $rank(A_{s+1}) = rank(I_{\beta,\beta}) = \beta = N_{s+1} - N_s$.

Computation the matrix rank of $F_t(2,2)$ network

Similar to the $F_t(1,3)$, let α be the set of nodes that are belong to the s-step network $F_t(2,2)$, $\alpha = N_s$; and β be the set of nodes that are generated at the $(s+1)$-th iteration, $\beta = N_{s+1} - N_s$. From the construction, at $(s+1)$-th step, the adjacency matrix $B_{s+1}(2,2)$ of the network $F_{s+1}(2,2)$ has the following form:

$$\begin{aligned} B_{s+1} &= \begin{pmatrix} B_{\alpha,\alpha} & B_{\alpha,\beta} \\ B_{\beta,\alpha} & B_{\beta,\beta} \end{pmatrix} \\ &= \begin{pmatrix} 0 & B_{\alpha,\beta} \\ B_{\beta,\alpha} & 0 \end{pmatrix} \\ &= \begin{pmatrix} 0 & B_{\alpha,\beta} \\ B^T_{\alpha,\beta} & 0 \end{pmatrix}. \end{aligned} \tag{6}$$

Then, we can obtain the rank of the adjacency matrix B_{s+1} as:

$$\begin{aligned} rank(B_{s+1}) &= rank\left(B_{\alpha,\beta}\right) + rank\left(B^T_{\alpha,\beta}\right) \\ &= 2rank(B_{\alpha,\beta}), \end{aligned} \tag{7}$$

where $B_{\alpha,\beta}$ is a matrix with N_s rows and $N_{s+1} - N_s$ columns as follows:

$$B_{\alpha,\beta} = \begin{pmatrix} b_{1,N_s+1} & b_{1,N_s+2} & \cdots & b_{1,N_{s+1}} \\ b_{2,N_s+1} & b_{2,N_s+2} & \cdots & b_{2,N_{s+1}} \\ \vdots & \vdots & \ddots & \vdots \\ b_{N_s,N_s+1} & b_{N_s,N_s+2} & \cdots & b_{N_s,N_{s+1}} \end{pmatrix}.$$

The labels of $B_{\alpha,\beta}$'s column are from $N_s + 1$ to N_{s+1}, and labels of $B_{\alpha,\beta}$'s row are 1 to N_s.

Below, we give the process how to calculate the rank of the matrix $B_{\alpha,\beta}$ in $F_t(2,2)$ network. According to the evolution of $F_t(2,2)$ network, each edge of $t = s$ can be generated two symmetrical new nodes at $t = s+1$, and the corresponding columns are identical in the adjacent matrix $B_{\alpha,\beta}$. Thus, we implement the elementary column transformation on the matrix B. Using the columns label as odd minus its adjacent columns label as even, every element is 0 of all columns labeled as even. By deleting these columns, the matrix $B_{\alpha,\beta}$ becomes a new matrix with α rows and $\frac{\beta}{2}(\frac{\beta}{2} = \frac{N_{s+1}-N_s}{2} = 2(N_s - N_{s-1}))$ columns at $t = s+1$, denoted by C_{s+1}. We have $rank(B_{\alpha,\beta}) = rank(C_{s+1})$.

In order to calculate the rank of C_{s+1}, we divide the C_{s+1} in to block form as following:

$$C_{s+1} = \begin{pmatrix} X & Y \\ Z & M \end{pmatrix}.$$

The forms of X ,Y, Z and M are as follows respectively.

X is a submatrix of C_{s+1}, it has N_{s-1} rows and $N_s - N_{s-1}$ columns, we give the form of X:

$$X_{N_{s-1},N_s-N_{s-1}} = \begin{pmatrix} 1\cdots 1 & 0\cdots 0 & \cdots & 0\cdots 0 \\ 0\cdots 0 & 1\cdots 1 & \cdots & 0\cdots 0 \\ \cdots & \cdots & \cdots & \cdots \\ 0\cdots 0 & 0\cdots 0 & \cdots & 1\cdots 1 \\ 0\cdots 0 & 0\cdots 0 & \cdots & 0\cdots 0 \\ \cdots & \cdots & \cdots & \cdots \\ 0\cdots 0 & 0\cdots 0 & \cdots & 0\cdots 0 \end{pmatrix}.$$

For the matrix X, the labels of rows with non-element are 1 to N_{s-2}, and elements of rest rows both are zero. In X, the number of 1 is 2^s in each row (from 1: N_1), and the number of 1 is 2^{s-i} in each row (form $N_i + 1$: N_{i+1}, $i = 1, 2, \cdots, s-3$).

Y is a submatrix has same size with X, its form is:

$$Y_{N_{s-1},N_s-N_{s-1}} = \begin{pmatrix} 0\cdots 0 & 0\cdots 0 & \cdots & 0\cdots 0 \\ \cdots & \cdots & \cdots & \cdots \\ 0\cdots 0 & 0\cdots 0 & \cdots & 0\cdots 0 \\ 1\cdots 1 & 0\cdots 0 & \cdots & 0\cdots 0 \\ 0\cdots 0 & 1\cdots 1 & \cdots & 0\cdots 0 \\ \cdots & \cdots & \cdots & \cdots \\ 0\cdots 0 & 0\cdots 0 & \cdots & 1\cdots 1 \end{pmatrix}.$$

In Y, there are non-element from $N_{s-2} + 1$-th row to N_{s-1} row, and the number of 1 is 2^2 in each row (form $N_{s-2} + 1 : N_{s-1}$).

Z is a identity matrix with $N_s - N_{s-1}$ rows and $N_s - N_{s-1}$ columns, its form as follow:

$$Z_{N_s-N_{s-1},N_s-N_{s-1}} = \begin{pmatrix} 1 & 0 & 0 & \cdots & 0 & 0 \\ 0 & 1 & 0 & \cdots & 0 & 0 \\ 0 & 0 & 1 & \cdots & 0 & 0 \\ 0 & 0 & 0 & \cdots & 0 & 0 \\ \cdots & \cdots & \cdots & \cdots & \cdots & \cdots \\ 0 & 0 & 0 & \cdots & 1 & 0 \\ 0 & 0 & 0 & \cdots & 0 & 1 \end{pmatrix}.$$

The matrix M can transform into the identity matrix which has the same size as Z. So, we have

$$M = \begin{pmatrix} 1\,0\,0\,0 & \cdots & 0\,0\,0\,0 & \cdots & 0\,0\,0\,0 \\ 0\,1\,0\,0 & \cdots & 0\,0\,0\,0 & \cdots & 0\,0\,0\,0 \\ 0\,0\,0\,0 & \cdots & 0\,1\,0\,0 & \cdots & 0\,0\,0\,0 \\ 0\,0\,0\,0 & \cdots & 0\,0\,1\,0 & \cdots & 0\,0\,0\,0 \\ 0\,0\,1\,0 & \cdots & 0\,0\,0\,0 & \cdots & 0\,0\,0\,0 \\ 0\,0\,0\,1 & \cdots & 0\,0\,0\,0 & \cdots & 0\,0\,0\,0 \\ \cdots & \cdots & \cdots & \cdots & \cdots \\ 0\,0\,0\,0 & \cdots & 0\,0\,0\,0 & \cdots & 1\,0\,0\,0 \\ 0\,0\,0\,0 & \cdots & 0\,0\,0\,0 & \cdots & 0\,1\,0\,0 \\ 0\,0\,0\,0 & \cdots & 0\,0\,0\,0 & \cdots & 0\,0\,1\,0 \\ 0\,0\,0\,0 & \cdots & 0\,0\,0\,0 & \cdots & 0\,0\,0\,1 \end{pmatrix}.$$

By appropriately exchanging some columns of Y and M, we have

$$C_{s+1} \sim \begin{pmatrix} X & Y' \\ I & I \end{pmatrix}. \tag{8}$$

By implementing the elementary transformation on the matrix (B3), it follows that:

$$C_{s+1} \sim \begin{pmatrix} 0 & M_{s+1} \\ I & 0 \end{pmatrix}, \tag{9}$$

where

$$M_{s+1} = \begin{pmatrix} X \\ Y' \end{pmatrix}.$$

Thus, $rank(C_{s+1}) = rank(I_{N_s - N_{s-1}}) + rank(M_{s+1}) = N_s - N_{s-1} + rank(M_{s+1})$. We need to calculate the rank of M_{s+1}.

According the forms of X and $Y^{'}$, we know that every two adjacency columns are identical in the matrix M_{s+1}, by deleting one of the column, it follows that:

$$\begin{pmatrix}
11\cdots1 & \cdots & 00\cdots0 & 0\cdots0 & 0\cdots0 & \cdots & 0\cdots0 \\
\cdots & \cdots & \cdots & \cdots & \cdots & \cdots & \cdots \\
00\cdots0 & \cdots & 11\cdots1 & 0\cdots0 & 0\cdots0 & \cdots & 0\cdots0 \\
00\cdots0 & \cdots & 00\cdots0 & 1\cdots1 & 0\cdots0 & \cdots & 0\cdots0 \\
\cdots & \cdots & \cdots & \cdots & \cdots & \cdots & \cdots \\
00\cdots0 & \cdots & 00\cdots0 & 0\cdots0 & 0\cdots0 & \cdots & 1\cdots1 \\
10\cdots0 & \cdots & 00\cdots0 & 1\cdots0 & 0\cdots0 & \cdots & 0\cdots0 \\
\cdots & \cdots & \cdots & \cdots & \cdots & \cdots & \cdots \\
00\cdots0 & \cdots & 0\cdots10 & 0\cdots0 & 0\cdots1 & \cdots & 0\cdots0 \\
00\cdots0 & \cdots & 0\cdots01 & 0\cdots1 & 0\cdots0 & \cdots & 0\cdots1
\end{pmatrix}$$

Easy to observe that the matrix M_{s+1} is identical with the matrix C_s. By implementing similar elementary transformation, the matrix M_{s+1} converts into:

$$C_s \sim M_{s+1} \sim \begin{pmatrix} 0 & M_s \\ I & 0 \end{pmatrix}.$$

Thus, $rank(C_s) = rank(I_{N_{s-1} - N_{s-2}}) + rank(M_s) = N_{s-1} - N_{s-2} + rank(M_s)$. Similarly, the M_s is identical with the matrix C_{s-1}. By continue implementing elementary transformation for M_s, we get $rank(C_{s-1}) = rank(I_{N_{s-2} - N_{s-3}}) + rank(M_{s-1}) = N_{s-2} - N_{s-3} + rank(M_{s-1})$. By repeating this iterative process until $M_2 = C_1$, we have

$$\begin{aligned}
rank(B_{\alpha,\beta}) &= rank(C_{s+1}) \\
&= N_s - N_{s-1} + rank(C_s) \\
&= N_s - N_{s-1} + N_{s-1} - N_{s-2} + rank(C_{s-1}) \\
&= N_s - N_{s-2} + N_{s-2} - N_{s-3} + rank(C_{s-2}) \\
&= \cdots \\
&= N_s - N_1 + N_1 - N_0 + rank(C_1) \\
&= N_s - N_0 + rank(C_1).
\end{aligned}$$

By the construction, we have $rank(C_1) = rank(B_{\alpha,\beta})$, where, $\alpha = 1, \beta = 1$. Since

$$B_{1,1} = \begin{pmatrix} 1 & 1 \\ 1 & 1 \end{pmatrix},$$

we have $rank(C_1) = rank(B_{1,1}) = 1$. It is easy to compute, $N_0 = 2$, hence, $rank(B_{\alpha,\beta}) = N_s - 2 + 1 = N_s - 1$. Then, by equation (B2), $rank(B_{s+1}) = 2(N_s - 1)$.
